# Supplementary figures and images for: Circulating adiponectin mediates the association between omentin gene polymorphism and cardiometabolic health in Asian Indians
Source: PLoS One. 2021 May 12;16(5):e0238555. doi: 10.1371/journal.pone.0238555 (PMC8115825; doi:10.1371/journal.pone.0238555)

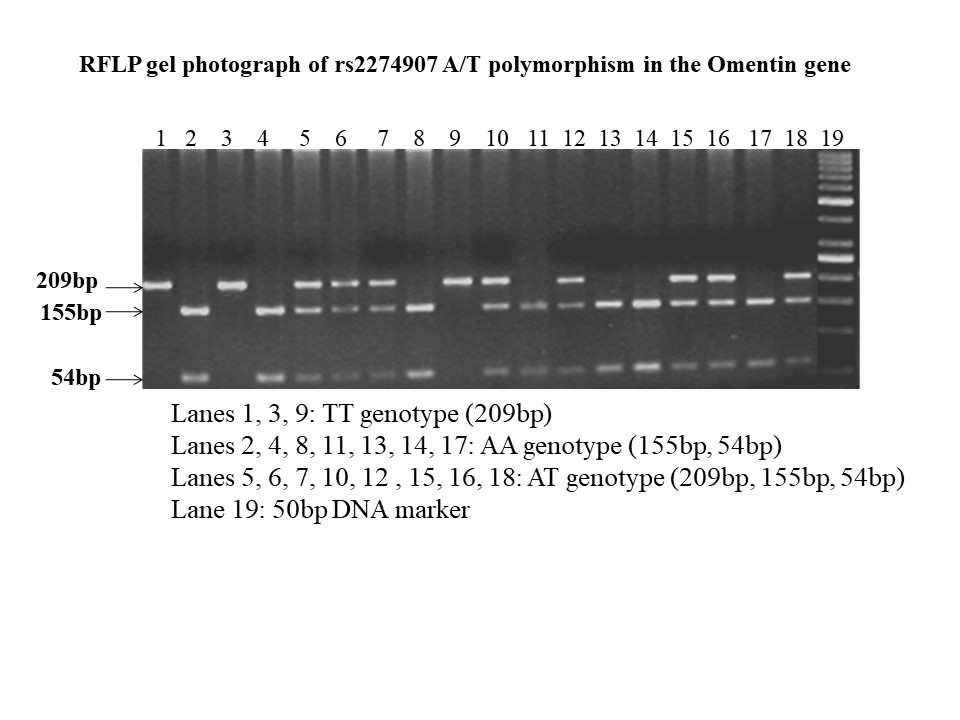

Supplement: S1 Fig — (TIF) [file pone.0238555.s001.tif]
