# Supplementary material for: Circulating adiponectin mediates the association between omentin gene polymorphism and cardiometabolic health in Asian Indians
Source: PLoS One. 2021 May 12;16(5):e0238555. doi: 10.1371/journal.pone.0238555 (PMC8115825; doi:10.1371/journal.pone.0238555)
Supplement: S1 Table — (DOCX) [file pone.0238555.s002.docx]

**Supplementary table 1: Association between the Omentin SNP rs2274907** **and cardiometabolic health status.**

| **Genotypes** | **Cardiometabolic health status** | | |
| --- | --- | --- | --- |
|  | **Cardiometabolically healthy (N=370)** | **Cardiometabolically Unhealthy (N=1,516)** | ***P-value**** |
| **Additive model** | | | |
| **TT** | 231  (62.4%) | 910  (60.0%) | 0.31 |
| **AT** | 127  (34.3%) | 530  (35.0%) |  |
| **AA** | 12  (3.2%) | 76  (5.0%) |  |
| **Minor allele frequency (T)** | 20.0% | 22.0% |  |
| **Dominant model** | | | |
| **TT** | 231  (62.4%) | 910  (60.0%) | 0.40 |
| **AT + AA** | 139  (37.6%) | 606  (40.0%) |  |

* P values from the Chi-square test for the differences in the genotype frequencies
